# From Radical Mastectomy to Breast-Conserving Therapy and Oncoplastic Breast Surgery: A Narrative Review Comparing Oncological Result, Cosmetic Outcome, Quality of Life, and Health Economy

**DOI:** 10.1155/2013/742462

**Published:** 2013-09-12

**Authors:** Ahmad Kaviani, Nassim Sodagari, Sara Sheikhbahaei, Vahid Eslami, Nima Hafezi-Nejad, Amin Safavi, Maryam Noparast, Alfred Fitoussi

**Affiliations:** ^1^Department of General Surgery, Tehran University of Medical Sciences (TUMS), P.O. Box 13145-158, Tehran, Iran; ^2^Kaviani Breast Disease Institute (KBDI), P.O. Box 14358-6443, Tehran, Iran; ^3^School of Medicine/Public Health, Tehran University of Medical Sciences (TUMS), P.O. Box 1416135, Tehran, Iran; ^4^Student's Scientific Research Centre (SSRC), Tehran University of Medical Sciences (TUMS), P.O. Box 14155-6537, Tehran, Iran; ^5^Sina Trauma and Surgery Research Centre, Tehran University of Medical Sciences (TUMS), P.O. Box 11365-3876, Tehran, Iran; ^6^The Young Scholars Club, No. 8 West Niloofar Street, Africa (Jordan) Avenue, Tehran, Iran; ^7^Iran's National Elite Foundation, No. 17 Haghighat Talab Street, Southern Nejatollahi Avenue, Tehran, Iran; ^8^Department of Surgery, Institute Curie, P.O. Box 75005, Paris, France

## Abstract

Surgical management of breast cancer has evolved considerably over the last two decades. There has been a major shift toward less-invasive local treatments, from radical mastectomy to breast-conserving therapy (BCT) and oncoplastic breast surgery (OBS). In order to investigate the efficacy of each of the three abovementioned methods, a literature review was conducted for measurable outcomes including local recurrence, survival, cosmetic outcome, quality of life (QOL), and health economy. From the point of view of oncological result, there is no difference between mastectomy and BCT in local recurrence rate and survival. Long-term results for OBS are not available. The items assessed in the QOL sound a better score for OBS in comparison with mastectomy or BCT. OBS is also associated with a better cosmetic outcome. Although having low income seems to be associated with lower BCT and OBS utilization, prognosis of breast cancer is worse in these women as well. Thus, health economy is the matter that should be studied seriously. OBS is an innovative, progressive, and complicated subspeciality that lacks published randomized clinical trials comparing surgical techniques and objective measures of outcome, especially from oncologic and health economy points of view.

## 1. Introduction

Until recently, surgical management of Breast Cancer (BC) has focused on two main choices: tumor resection using breast conserving therapy (BCT) and mastectomy with optional tissue displacement by breast reconstruction. From 2003, the techniques that combine the skill of resection and reconstruction in one procedure were introduced that can be named as the third approach, oncoplastic breast surgery (OBS). This approach involves reconstruction of the resection defects by volume displacement using adjacent breast tissue. Both techniques are adopted from the conventional methods of breast reduction and reconstruction [1].

Mastectomy includes excision of the breast tissue and is divided into subtypes according to the resection of lymph nodes and muscles. Traditionally, it is employed when the risk of local recurrence is increased by tumor size of greater than 5 cm, presence of lymphovascular invasion, presence of more than 4 suspected nodes, and involved or closed margins [2].

BCT is composed of lumpectomy or wide local excision and axillary dissection with or without radiotherapy. It was accepted as a surgical option after thorough evaluation in six international prospective randomized trials for early stages of BC (Stages I and II) [3, 4]. Traditional contraindication to perform BCT includes large tumor size (>5 cm), skin or chest wall involvement, multicentric tumors, or anticipated poor cosmetic outcome, and whenever radiation therapy is contraindicated [5].

OBS unites large lumpectomy and remodelling procedures and involves different plastic surgery methods. OBS improves the cosmetic results after partial mastectomy and widens the possibility of conservative treatment. Today these techniques are well codified. They range from simple reshaping to more complicated techniques involving concomitant contralateral breast symmetrisation procedures [6].

As in many solid tumors, when facing BC, patients and surgeons are focused on three concerns: survival, local control, and quality of life. In this review article we compare mastectomy, BCT, and OBS to elucidate the current literature consent from four points of view, including (1) oncological result, (2) cosmetic outcome, (3) quality of life (QOL), and (4) health economy.

## 2. Methods

A computerized MEDLINE search was performed for English language abstracts and English and French articles published between January 2000 and June 2013 on “Mastectomy,” “Breast Conserving Therapy,” and “Oncoplastic Breast Surgery.” The headings were combined with the words including “Oncological Result,” “Local Recurrence,” “Survival,” “Cosmetic Outcome,” “Quality of Life,” and “Health Economy.” Other papers were identified from the bibliographies of these articles. More than 60 articles were reviewed. Of note, studies comparing QOL between mastectomy and BCT that were published before 2008 have been reviewed by Pockaj et al. previously [7]. Finally a narrative review was constructed to summarize the comparisons of the three modalities in the four identified perspectives by the expert authors.

## 3. Results and Discussion

### 3.1. Oncological Results, Local Recurrence, and Survival

It sounds that the oncological result is the most important part of any procedure done for the BC treatment. Oncological results are mostly investigated in two fields: local recurrence and survival. Although radical and modified radical mastectomies are both effective, they accompanied a huge psychological burden. Therefore, conservative procedures were put forward as alternative choices [8]. Studies demonstrate that the overall disease-free survival from BC is equivalent for mastectomy and BCT along with postoperative radiotherapy; especially in women with early stage BC (Stages I and II) [8]. BCT can be performed in larger tumors as compared to lumpectomy [9]. OBS may have an inferior long-term oncological result in comparison [10]. Next we discuss the local recurrence and the survival status associated with each of the BC surgical modalities.

#### 3.1.1. Local Recurrence

In mastectomized patients, a 2–10% chest wall recurrence has been reported. Local recurrence rate after BCT is 3–17% [8]. Majority of patients with recurrence after BCT can be salvaged with mastectomy. Additional local recurrence after salvage mastectomy has been reported in nearly 5% of patients [8]. So, the national institute of health recognized the equivalency of medical outcome for these two procedures in a consensus development conference statement in 1990 and recommended BCT as an appropriate method of primary therapy for most women with early stage BC [3, 8, 11].

There are factors associated with local recurrence after BCT such as young age, positive resection margins (for both mastectomy and BCT), multicentric disease, vascular invasion, high-grade tumor, necrosis tumor, infralymphatic extension, and extensive intraductal component. There are controversial opinions on the optimal extent of resection to obtain tumor-free margins including 2 mm negative microscopic margin versus 1-2 cm macroscopic uninvolved tissue. It seems, that with breast irradiation, as a part of BCT, wide tumor-free margins have little practical importance [8]. On the other hand, some studies show that the surgical margin significantly affects the 10-year disease-free survival rate and is the most important factor to reduce the risk for local recurrence [12].

Choosing the right patient for BCT is an important matter to be noted. Many factors contribute to this decision such as patient's desire; as she should undergo daily outpatient radiotherapy over 5-6 weeks to a total dose of 50 Gy. Other factors include size of the breast (small breasts are not good candidate for BCT) and tumor size of less than 2.5–5 cm (tumors larger than 5 cm are consistent with likelihood of local recurrence). Also, there are few cases in which BCT is contraindicated for concerns of toxicity or marked risk of local recurrence. Pregnancy in the first or second trimester is an absolute contraindication. Multifocal disease (two or more gross lesions in different quadrants or diffuse microcalcification on mammography), prior radiation therapy, and rheumatologic disorders are relative contraindications to BCT. We recall that BCT without radiotherapy has a local recurrence rate of 6–45%. Many experts believe that oncological result is the priority which shall not be exchanged with morphological symmetry and cosmetic outcomes [10].

Local recurrence rate after OBS has not thoroughly studied yet. Most of the available studies on OBS have shortcomings in their methodology which undercuts their conclusive robustness [13]. However, a number of studies showed 0–7% local recurrence rate after up to 54 months of followup [14–31]. Studies on the long-term outcome after OBS are not available, but the local recurrence rate of 0–1.8% per year for OBS on intermediate followup suggests that these techniques are associated with low local recurrence rates. New studies have recommended OBS as a probable approach for all women undergoing mastectomy for BC [32]. 

#### 3.1.2. Survival

There are no significant differences between BCT with radiotherapy and mastectomy regarding the overall survival rates (40–95% in five years) [33]. Until now, no prospective randomized or retrospective trial study, comparing the survival rate between BCT and OBS, has been performed. Nevertheless, authors of noncomparative studies have reported that OBS results in the same survival rate [34]. We should consider that OBS techniques commonly render the possibility of a simple further excision. The difficulty of the procedure leaves many patients with involved margins who need further mastectomy, while mastectomy can be avoided in patients with BCT [35].

### 3.2. Cosmetic Outcome

Aesthetic surgery of healthy breasts has become a major industry over the past half century. Some of the indications have medical basis, but much of the demand has arisen from more complex psychosocial, economic, and reproductive pressure of human male to human female. Developments in aesthetic breast surgery have encouraged considerable experimentation in immediate or delayed symmetrisation at cancer surgery [36].

Cosmetic reconstruction should not follow geometric patterns but should emphasize on perceived contour and normal clothing lines. One goal of breast reconstruction is to restore the breast as normally and as attractively while minimizing visible scars [20]. The understandable advantages of BCT are equivalent local and distant control rates as compared with mastectomy. But the key to a successful BCT is achieving a cosmetic outcome that is acceptable to the patient and the physician. Cosmetic outcome is the end result of a range of factors which come together under a broad head of surgery, radiotherapy, chemotherapy, and hormonal treatment [37]. On the other hand, feeling of disfigurement, mutilation, and insult to femininity are the matter to be feared in modified radical mastectomy [37, 38]. It is obvious that the comparison between mastectomy without reconstruction and the other two methods (BCT and OBS) is not meaningful. Therefore, we discuss the cosmetic outcome in BCT and OBS.

Various criteria have been employed in different studies to compare the cosmetic outcome [40]. In Breast Cancer Treatment and Outcome Survey (BCTOS), a 22-item survey instrument was designed to measure the perceived aesthetic and functional status after BCT and radiotherapy. It was further validated in a BC population [38]. For instance, aesthetic triangle was defined by the sternal notch-nipple (SN-N) and nipple-nipple parameters [40]. Numbers of innovating grading systems were introduced by panels of surgeons and nonmedical personnel [15, 37]. Briefly, a good aesthetic result nearly means little or no residual asymmetry, minor postradiotherapy after-effects and no dimpling [6].

Investigators had classified breast asymmetry risk factors in BCT into two groups: patient-related and treatment-related factors. Patient-related risk factors include young age (since breast and nipple areolar complex lay progressively infero-lateral), high BMI, large tumor size, and resection of superior-medial and inferior-lateral tumors. The allowed volume reduction is 5% in medial, in comparison to 15% in lateral. Treatment-related risk factors for asymmetry contain reexcision lumpectomy, postoperative seroma and radiation therapy [37]. Extremely small sized breasts and tumor sizes greater than 5 cm may have an unacceptable cosmetic result [8].

Our findings show that the cosmetic result of BCT is not always satisfactory to the patients or the surgeons and OBS seems to provide a more promising future [15, 37, 38, 40]. Cosmetic failure rates after BCT are approximately 30%; while this rate is 0–18% for OBS [14–19, 21–31, 40]. Selecting the appropriate patient according to the known risk factors can prevent frankly poor results in 25–30% of BCTs [40]. This includes absolute indications for mastectomy, containing widespread DCIS, multifocal tumor, and recurrence. By progressions in the treatment of BC, patients prefer to be treated well even by mastectomy rather than choosing a pure aesthetical satisfactory BCT [40]. 

Given that different techniques of OBS have been introduced in recent years, the investigations on aesthetic outcomes after OBS are very few. According to these few studies it seems that there are some important advantages for OBS in comparison with the other traditional surgeries. First of all, these techniques have reduced the worries of incomplete excision for surgeons. A very large excision can be taken with OBS along with a good cosmetic result and least deformity. Secondly, the cosmetic outcome is affected considerably by radiation therapy. In OBS the effect of radiation therapy as an insult for cosmetic outcome gets the minimal level. This is achieved by refilling the cavity of excision by adjacent breast tissue. Although OBS techniques generally produce larger scars, postoperative irradiation invariably improves their appearance [40]. Timing of radiotherapy, beam intensity, boost area, volume, and dose are all important in the cosmetic outcome [37]. The last but not the least important point refers to the fact that the amount of safe margin and the need for reoperation have been reduced in patients operated by these newer techniques. 

The limitation of these surveys is that direct comparison between these studies is difficult and also the methods vary both in regard to the surgery and to the cosmetic assessment [15]. Role of plastic surgery in surgical treatment of BC has increased during the last two decades and this trend is likely to continue during the next decade [11, 36]. Therefore, we recommend a multicentral, longitudinal, and prospective study to standardize a cosmetic outcome assessment and to clarify the indication of each technique.

### 3.3. Quality of Life

BC is a common disease with a relatively good prognosis especially when detected in early stages. The number of BC survivors is both very large and increasing. Thus, QOL in BC survivors has been an important topic [4]. Improvement in QOL can motivate more women to follow screening programs for early diagnosis of the disease. Moreover, QOL has a major role in decision making of patients and physicians [41].

Vast medical literature is available on the QOL of patients who underwent mastectomy and BCT. However, lack of studies on this issue regarding OBS is evident. QOL is a subjective tool to measure common perception of patients regarding a number of aspects including body image, satisfaction, attractiveness, feeling whole, subjective cosmetic results, appearance, scar, insecurity, sexual problems, postsurgical complications such as lymphedema, arm swelling, limitation in range of motion, psychiatric distress, anxiety, depression, and worrying about the future. These aspects are associated with other factors like chemotherapy, medical condition, social support, income, education, and so on [4, 7, 41–46]. For such purposes, studies inquired recognized questionnaires like European Organization for Research and Treatment of Cancer (EORTC), Cancer Rehabilitation Evaluation System—Short Form (CARES-SF), Short Form Questionnaire (SF-30), Quality of Life Questionnaire (QLQ-C30), and Hospital Anxiety and Depression Scale (HADS). In some articles there were de novo questionnaires as well.

Randomized clinical trials (RCTs) have shown no difference in prognosis and survival between BCT and mastectomy [42, 47, 48]. Two of the six international trials reported their 20-year followups confirming equal long-term survival and local control with mastectomy when compared to BCT [3, 47, 49]. In this regard Pockaj and colleagues comprehensively reviewed medical literature to investigate the QOL outcome and factors that influence these outcomes in women undergoing various BC surgical procedures. Summing the results of various studies, we found that QOL assessment is divided into two groups: short term and long-term after the procedure. It seems that long-term evaluation of QOL with famous questionnaires is more validated [4, 7, 42]. It is a common belief that if surgical effect could be established with less-extensive surgery, cosmetic outcome would be improved and the QOL would be maintained with minimal attenuation in functional aspects of the operated areas. Many small sample-sized RCT surveys showed that the initially expected results of excellent QOL in BCT patients have not been definitely shown except for body image (attractiveness, appearance, feeling whole, cosmetic outcome, scar, and insecurity) in comparison with mastectomy or even breast reconstruction after mastectomy [4, 7, 43, 50, 51]. However, few studies which had long-term evaluations after the operation showed either a statistically significant different [52] or no different results [53, 54].

In one of these studies, although there was no statistically significant difference in QOL between the two groups, among the patients who were younger than 50 years at the time of diagnosis, BCT appeared to be protective against psychiatric distress when compared with total mastectomy. Patients of 50 years of age or older at the time of diagnosis who received BCT had higher psychological stress level about fearing of recurrence and deficit of treatment [53].

Some nonrandomized clinical trials declared that being a younger patient with superior socioeconomic status, higher educational background, stable marital relationship, and using BCT is linked to a better QOL [4, 43] and sexuality [44]. On the other hand, most studies showed that mastectomized patients scored constantly worse in the variables associated with body image and were less satisfied with their cosmetic outcome and were more likely to choose a different surgical treatment if they had a chance to do it over again. Of note, they had more physical symptoms and discomfort around the surgical site [4, 7]. So we propose that BCT carries a lower complication rate, a shorter operative time, an improved body image, and decreased insecurities in intimate relations in comparison with mastectomy. Actually, the increasing rate of BCT in all age groups is of the major quality indicators as well [3, 55].

OBS is an issue of debate, mostly because of its novelty [56]. The percept that immediate breast reconstruction improves health-related QOL underlines the increasing practice of breast reconstruction, internationally [57]. However, we should take into consideration that the longer time of surgery, the more fat necrosis in obese patients in some type of reduction mammoplasty, and the poor cosmetic outcome in 5–14% of OBSs necessitates more RCT studies in this field [35]. Many superior aspects of OBS will decline in the long term [58]. Nevertheless, newer studies indicate OBS as a safe and effective method even as a delayed surgery [59].

### 3.4. Health Economy

We did not find any study directly comparing mastectomy, BCT, and OBS from the view of health economy. Previous studies insist on the restricted access of low income women to radiotherapy after BCT. This fact places them at an increased risk of having worse outcome in comparison to those treated with standard mastectomy [60, 61]. Besides, lower proportion of BCT and OBS is performed among the patients without insurance or medicaid support. The presentation of BC is worse in these women as well [62].

There was no published article regarding the health economy of OBS. It sounds as if plastic surgery all over the world is an expensive operation, but, in comparison with mastectomy and BCT, one should consider all the after effects before declaring the most economically advantageous method [63]. OBS may reduce the need for mastectomy and expensive breast reconstruction surgeries [64]. We note a prompt need for economic evaluation studies to be performed in this field. 

## 4. Conclusion

Aesthetic outcome is one of the major concerns of patients and physicians in BC surgery [39]. Breast conservation techniques are employed more and more even in the oldest age groups [55]. OBS has a positive impact on all aspects of QOL [57]. High patient satisfaction and proven oncological safety are the advantages of OBS when compared to BCT and mastectomy [32]. When performed by trained specialists, OBS accompanies a favourable outcome even in settings with inferior resources [64]. However, the superiority of patient's administration of cosmetic outcome and QOL may decline in long followup [58]. The long-term results of OBS are yet to be understood [13] and the traditional procedures may be more promising in their oncological outcome [10]. Breast surgeon has an important role in patient's decision making [63]. Proper patient selection and careful planning are of great importance to achieve an acceptable result [56, 59].
